# Association of Low-Dose Whole-Body Computed Tomography With Missed Injury Diagnoses and Radiation Exposure in Patients With Blunt Multiple Trauma

**DOI:** 10.1001/jamasurg.2019.5468

**Published:** 2020-01-15

**Authors:** Dirk Stengel, Sven Mutze, Claas Güthoff, Moritz Weigeldt, Konrad von Kottwitz, Domenique Runge, Filip Razny, Anna Lücke, Dirk Müller, Axel Ekkernkamp, Thomas Kahl

**Affiliations:** 1 Center for Clinical Research, BG Klinikum Unfallkrankenhaus Berlin gGmbH, Berlin, Germany; 2 Department of Trauma and Orthopaedic Surgery, BG Klinikum Unfallkrankenhaus Berlin gGmbH, Berlin, Germany; 3 BG Kliniken–Klinikverbund der Gesetzlichen Unfallversicherung gGmbH, Berlin, Germany; 4 Institute of Radiology, BG Klinikum Unfallkrankenhaus Berlin gGmbH, Berlin, Germany; 5 Consultant in radiation physics, Hamburg, Germany

## Abstract

**Importance:**

Initial whole-body computed tomography (WBCT) for screening patients with suspected blunt multiple trauma remains controversial and a source of excess radiation exposure.

**Objective:**

To determine whether low-dose WBCT scanning using an iterative reconstruction algorithm does not increase the rate of missed injury diagnoses at the point of care compared with standard-dose WBCT with the benefit of less radiation exposure.

**Design, Setting, and Participants:**

This quasi-experimental, prospective time-series cohort study recruited 1074 consecutive patients admitted for suspected blunt multiple trauma to an academic metropolitan trauma center in Germany from September 3, 2014, through July 26, 2015, for the standard-dose protocol, and from August 7, 2015, through August 20, 2016, for the low-dose protocol. Five hundred sixty-five patients with suspected blunt multiple trauma prospectively received standard-dose WBCT, followed by 509 patients who underwent low-dose WBCT. Confounding was controlled by segmented regression analysis and a secondary multivariate logistic regression model. Data were analyzed from January 16, 2017, through October 14, 2019.

**Interventions:**

Standard- or low-dose WBCT.

**Main Outcomes and Measures:**

The primary outcome was the incidence of missed injury diagnoses at the point of care, using a synopsis of clinical, surgical, and radiological findings as an independent reference test. The secondary outcome was radiation exposure with either imaging strategy.

**Results:**

Of 1074 eligible patients, 971 (mean [SD] age, 52.7 [19.5] years; 649 men [66.8%]) completed the study. A total of 114 patients (11.7%) had multiple trauma, as defined by an Injury Severity Score of 16 or greater. The proportion of patients with any missed injury diagnosis at the point of care was 109 of 468 (23.3%) in the standard-dose and 107 of 503 (21.3%) in the low-dose WBCT groups (risk difference, −2.0% [95% CI, −7.3% to 3.2%]; unadjusted odds ratio, 0.89 [95% CI, 0.66-1.20]; *P* = .45). Adjustments for autocorrelation and multiple confounding variables did not alter the results. Radiation exposure, measured by the volume computed tomography dose index, was lowered from a median of 11.7 (interquartile range, 11.7-17.6) mGy in the standard-dose WBCT group to 5.9 (interquartile range, 5.9-8.8) mGy in the low-dose WBCT group (*P* < .001).

**Conclusions and Relevance:**

Low-dose WBCT using iterative image reconstruction does not appear to increase the risk of missed injury diagnoses at the point of care compared with standard-dose protocols while almost halving the exposure to diagnostic radiation.

## Key Points

**Question:**

Is low-dose whole-body computed tomography with statistical image reconstruction associated with similar rates of missed injuries and accuracy but reduced radiation exposure compared with standard-dose whole-body computed tomography in the primary diagnostic workup of blunt multiple trauma?

**Findings:**

In this quasi-experimental cohort study of 971 patients with suspected blunt multiple trauma, participants in the standard-dose and low-dose whole-body computed tomography groups had the same risk of missed injury diagnoses. Low-dose scanning markedly reduced exposure to radiation, improved the contrast-to-noise ratio, and showed similar diagnostic accuracy among the investigated anatomical areas and organs when compared with standard-dose scanning.

**Meaning:**

These findings suggest that low-dose whole-body computed tomography may safely replace standard-dose scanning in the primary diagnostic workup of blunt multiple trauma.

## Teaser

This time-series cohort study assesses whether low-dose whole-body computed tomography increases the rate of missed injury diagnoses at the point of care compared with the standard dose among patients with multiple trauma.

## Introduction

In industrialized countries, mortality and disability-adjusted life-years attributable to blunt multiple trauma decreased markedly during the past decades.^1^ Advanced Trauma Life Support (ATLS),^2,3^ implementation of trauma centers and networks,^4^ hemostatic resuscitation,^5,6^ early pelvic stabilization,^7^ point-of-care ultrasonography,^8^ resuscitative endovascular balloon occlusion of the aorta,^9^ and other complex interventions contributed to this trend.^10^ Controversy exists about contrast-enhanced, whole-body computed tomography (WBCT) as a primary screening modality for suspected multiple trauma.^11,12,13,14^ Whole-body computed tomography shows excellent specificity but varying sensitivity for diagnosing injuries to different body areas.^15^ Apart from potential advantages on process quality, WBCT bears the risk of excessive exposure to diagnostic radiation.^16,17,18^

Modern scanner hardware and dose-sparing protocols have decreased radiation exposure with WBCT and, thus, the lifetime attributable risk of cancer.^16,19^ Recent noise-reducing image processing techniques, such as adaptive statistical iterative reconstruction (GE Healthcare) and iDose^4^ (Philips Healthcare), may further limit the radiation dose and likelihood of biological damage.^17,20^ In this study, we hypothesized that low-dose WBCT does not increase the risk of missed injury diagnoses at the point of care compared with standard-dose WBCT for screening patients with suspected blunt multiple trauma while exposing them to much less radiation.

## Methods

### Study Design and Setting

This prospective time-series cohort study (Dose Reduction in Whole-Body Computed Tomography of Multiple Injuries [DoReMI]) was conducted at an academic urban trauma center in Berlin, Germany, accredited by the German Society for Trauma Surgery. The DoReMI study enrolled patients with suspected blunt multiple trauma scheduled for initial WBCT. This study was approved by the institutional review board (IRB) of the Charité Universitätsmedizin, Berlin, Germany, in November 2013. The IRB approved inclusion of unconscious and ventilated, hemodynamically unstable patients, conditional on establishing pathways to obtain written informed consent from the individual patient or the patient’s next of kin or legal representative. This study followed the Strengthening the Reporting of Observational Studies in Epidemiology (STROBE) reporting guideline.

### Selection of Participants

Male and female patients of all ages with suspected blunt multiple trauma presenting to the emergency department and assigned to WBCT according to red flag criteria of the national evidence- and consensus-based best practice guideline for managing patients with severe injuries (Box) were eligible to participate in the study.^21^ Indication for WBCT matched international recommendations as summarized in recent systematic reviews.^22,23,24,25,26^ Patients were approached, informed about the study, and asked for consent to participate as soon as allowed by their physical and mental condition. Relatives, legal representatives, and patients were informed by physicians or professional clinical trial staff.

Box. **Inclusion and Exclusion Criteria**

**Inclusion Criteria**

Suspected blunt multiple trauma resulting from the following:
  Car crash with extrication or death of ≥1 occupant
  Automobile vs pedestrian or bicycle
  Fall from a height greater than 10 ft (3 m)
  Any other high-velocity injury mechanism
Resuscitation on scene or at the trauma bay by a multiprofessional team of paramedics and emergency physicians (including sedation or general anesthesia and airway management by orotracheal intubation)

**Exclusion Criteria**

Patients considered unsuitable for WBCT for any reason (eg, need for immediate life-saving thoracotomy, laparotomy, or cranial trepanation before imaging)
Patients declared dead on arrival or did not survive CPR
Patients not allowed to take part because of refusal by their relatives or legal representatives

Abbreviations: CPR, cardiopulmonary resuscitation; WBCT, whole-body computed tomography.

### Intervention

All patients were managed according to Pre-Hospital Trauma Life Support and ATLS principles by certified health care professionals or instructors, as mandated by the American College of Surgeons and the German Society for Trauma Surgery. After admission to the emergency department, patients were treated by an interdisciplinary team of trauma surgeons, anesthesiologists, radiologists, and nurses. This treatment included damage-control resuscitation, surgeon-performed focused ultrasonography of the thorax and abdomen, intubation and ventilation, relief of pneumothorax by chest tubes, and placement of central intravenous lines. At the investigational site, the CT suite is located opposite to the trauma bay. Patients were assigned to damage control or definitive surgery, intensive care, admission to a general trauma ward, or discharge and ambulatory care, according to their individual injury severity and pattern.

All WBCT examinations were performed on a 128-row scanner (Philips Ingenuity Core; Philips Healthcare) in the standard-dose and low-dose study periods. All hardware and scanning protocols (including the iDose^4^ image processing algorithm) were approved by the US Food and Drug Administration and the European Medicines Agency.

Patients were placed supine on the CT table with arms at their side. A low-dose scout imaging routine (Surview; Philips Healthcare) was followed for individual adjustment of scanning parameters. A native scan of the skull and midface was performed first, followed by contrast-enhanced whole-body imaging from the cranial base to the pelvis at the trochanteric level (eTable 1 in the Supplement). Automatic bolus tracking (BolusPro; Philips Healthcare) was used to trigger scans by placing a region of interest in the ascending aorta. Scanning was initiated 30 seconds after a predefined threshold of 120 Hounsfield units was reached. Images were stored in the local picture archiving and communication system (IntelliSpace PACS; Philips Healthcare).

During the low-dose period, the iDose^4^ hybrid iterative reconstruction algorithm was used in adjunct to optimized tube energy and effective output. iDose^4^ is supposed to reduce noise and overcome inherent limitations of filtered back projection while maintaining the quality and usual clinical impression of CT images.^27,28^

### Data Collection

Data were collected from September 3, 2014, through August 20, 2016. All initial WBCT scans were read by board-certified radiologists, and findings were immediately reported to the trauma team in the initial (“hot”) report. For quality ascertainment, all primary scans were reread by the radiological consultant on call at the earliest opportunity, and results were presented during the next interdisciplinary morning (7:45 am) or afternoon (2:30 pm) trauma rounds.

The local trial coordinating unit was responsible for data management, including data entry, plausibility checks, and query generation. Data were entered, stored, and processed using an electronic data capture system (secuTrial; interActive Systems). Data management complied with recent European General Data Protection Regulation.

Injuries were classified according to the Abbreviated Injury Scale coding scheme issued by the Association for the Advancement of Automotive Medicine as incorporated in recommendations by the American College of Surgeons and trained during ATLS courses. Patients enrolled during the standard-dose and low-dose periods were followed up in a similar fashion. A clinical synopsis using surgical reports and subsequent radiological examinations was used as an independent reference test to identify false-negative findings (missed diagnoses at the point of care) and false-positive findings (ie, injuries suspected by initial reading that could not be reproduced during follow-up). After discharge, any information about outpatient visits, reports by rehabilitation facilities, and private practice health care professionals was collected to identify false-negative or false-positive findings of the initial WBCT examination.

Because lethal injuries are considered nonnatural causes of death in Germany, corpses of injured patients are confiscated by prosecution officers. However, forensic autopsies for research purposes are difficult to obtain.

### Outcome Measures

A missed injury diagnosis at the point of care was defined as any injury demanding clinical awareness or therapeutic action at any time but that was not recognized in the initial WBCT or contained in the hot report provided to the trauma team. These diagnoses may be revealed during a second independent reading of the original scan. The DoReMI study was initiated ahead of the Berlin definition of multiple trauma incorporating physiological parameters in addition to an anatomical organ injury scale score.^29^ Multiple trauma was then indicated by the presence of injuries to 2 or more body regions that, alone or in combination, were life threatening or resulted in an Injury Severity Score of greater than 15.^30^

False-negative and false-positive findings may be caused by misinterpretation by the first reader or technical limitations to detect injuries. In the real world, it makes no difference whether a certain injury was missed or falsely presumed because of the reader’s fault or technical reasons, and the initial diagnostic information provided to the trauma team was accepted as the index test finding.

Dosimetry indexes were calculated automatically using section thickness, number of sections, and dose.^31^ This calculation included the volume CT dose index, dose-length product (DLP), and size-specific dose estimate. The CT dose index represents the energy dose (in milligrays) absorbed in a presumed rectangle profile of a single section with thickness provided by the manufacturer. The DLP accounts for the length of the scan, expressed as milligrays per centimeter. The size-specific dose estimates are adjusted for individual patient sizes and expressed as milligrays.

Subjective image quality among different tissues and sites was graded by 2 independent radiologists (F.R. and T.K.) using a 100-mm visual analog scale, with 0 indicating worst and 100 indicating perfect quality. Ratings were made in a paper-based fashion and subsequently entered in the electronic data capture system.

Objective image quality was assessed by the contrast-to-noise ratio.^32^ Standard regions of interest were placed in 7 anatomical landmarks: carotid artery, aortic arch, liver parenchyma, kidney cortex, abdominal aorta, cervical spine vertebra (C7), and lumbar spine vertebra (L1). The reference region of interest was placed in muscle tissue adjacent to the individual landmark on the same cross-sectional image.

### Statistical Analysis and Sample Size Calculation

Data were analyzed January 16, 2017, through October 14, 2019. At the time of planning and commencing this study, 3 previous investigations^15,33,34^ had specifically determined the risk of missed injuries with standard-dose WBCT in major trauma (including 1534 patients, 186 of whom had missed injuries). The pooled overall risk (using a random-effects model because of significant heterogeneity across studies, implemented in the STATA metaprop module [StataCorp LLC]) was 21% (95% CI, 7%-35% [eFigure 1 in the Supplement]). No reliable prior evidence was available to define noninferiority margins in categorical end points between the specific interventions of interest. Because of the quasi-experimental design of this study and lack of noninferiority margins, we did not use inferential statistics to test for noninferiority of low-dose compared with standard-dose WBCT. We attempted high precision of estimates and tight confidence intervals to substantiate our primary objective that low-dose WBCT does not increase the risk of missed injury diagnoses in a clinically meaningful or statistically significant manner compared with standard-dose WBCT.^35^ We aimed at a cohort size in which upper 97.5% binomial-exact confidence limits of the risk difference (RD) did not exceed 5% of the point estimate, given a baseline risk of missed injuries of 20% and varying scenarios with RDs of 4%, 2%, and 1% (eFigure 2 in the Supplement). This was guaranteed with a minimum sample size of 450 evaluable patients per group. To account for postallocation dropouts because of lack or denial of informed consent, missing data, and other sources of information loss, we aimed at enrolling 500 consecutive patients each in the standard-dose and low-dose WBCT periods. This approach was approved by the IRB and stated in the published trial protocol.^32^

Results are presented as means, medians, proportions, differences in means and proportions, RDs, and odds ratios (ORs), including measures of distribution and precision, such as SD, interquartile range (IQR), and 95% CI. The primary outcome, the proportion of patients with at least 1 missed injury diagnosis, was analyzed by a segmented regression model.^36^ This model assumes that, apart from seasonal fluctuation of events as operationalized by time intervals (ie, months), there is no bias by confounding variables. Autocorrelation was assessed by the Durbin-Watson statistic. We performed a secondary multivariate logistic regression analysis, adjusting OR of missed injury diagnoses at the point of care for age, sex, intubation, shock, coagulation measures, positive findings of focused ultrasonography of the thorax and abdomen, and the interval from admission to WBCT.

Prevalence, sensitivity, and specificity for excluding and confirming injuries with 95% CI were calculated using cross-tables. Complications, adverse events, and serious adverse events were recorded for either protocol. Medians and skewed continuous measures were compared by the Kruskal-Wallis test.

Reliability of subjective ratings was assessed by the intraclass correlation coefficient using a 2-way random-effects model. Substantial reliability was assumed with an intraclass correlation coefficient of 0.4 or higher for all 7 rated areas. We used SPSS, version 25.0 (IBM Corporation) and STATA, version 14.2 (Stata Corp LLC) for statistical analysis. *P* values were used with 95% CIs in an explanatory fashion.

## Results

**Figure 1.**
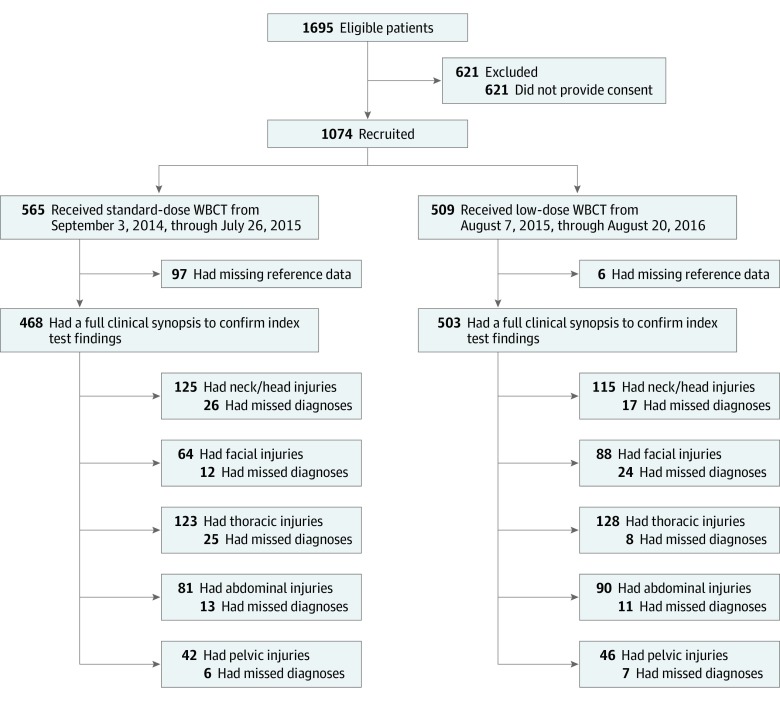
Study Profile and Flowchart WBCT indicates whole-body computed tomography.

Of 1695 patients screened, 1074 patients or their legal representatives consented to participate in the study. From September 3, 2014, through July 26, 2015, 565 patients underwent standard-dose WBCT, followed by 509 patients undergoing low-dose WBCT from August 7, 2015, through August 20, 2016. Altogether, data from 971 patients were available for primary end point analysis, with losses due to lacking information to compute a reference test (Figure 1). Among the 971 patients included in the analysis, mean (SD) age was 52.7 (19.5) years; 649 (66.8%) were men and 322 (33.2%) were women. One hundred fourteen patients (11.7%; 95% CI, 9.8%-13.9%) had multiple trauma. Baseline demographics for the 2 groups were similar (Table). Patients in the low-dose protocol period had a marginally higher mean (SD) international normalized ratio (1.3 [0.7] vs 1.2 [0.4]) and maximal Abbreviated Injury Scale score of the abdomen (2.4 [0.8] vs 2.1 [0.7]) and pelvis (2.4 [0.9] vs 2.2 [0.8]). Ultimate consequences from WBCT imaging and disposition of patients from the emergency department are shown in eTable 2 in the Supplement.

**Table.** Patient Characteristics

Characteristic	WBCT Group		*P* Value
	Standard-Dose (n = 468)	Low-Dose (n = 503)	
Age, mean (SD), y	52.9 (18.9)	52.5 (20.0)	.78
Male sex, No. (%)	312 (66.7)	337 (67.0)	.95
Mechanism of injury, No. (%)			
MVC	117 (25.0)	120 (23.9)	.81
Motorcycle	67 (14.3)	69 (13.7)	
Fall	198 (42.3)	200 (39.8)	
Auto vs pedestrian	22 (4.7)	28 (5.6)	
Cyclist	31 (6.6)	38 (7.6)	
Assault	7 (1.5)	10 (2.0)	
Other	26 (5.6)	38 (7.6)	
Transport or transfer, No. (%)			
Helicopter	123 (26.3)	141 (28.0)	.06
Ground ambulance			
Paramedics	116 (24.8)	134 (26.6)	
Physician staff	194 (41.5)	200 (39.8)	
Walk-in	25 (5.3)	27 (5.4)	
Other	10 (2.1)	1 (0.2)	
Orotracheal intubation, No. (%)	95 (20.3)	94 (18.7)	.57
Shock index, mean (SD)^a^	0.7 (0.4)	0.7 (0.3)	.97
Hemoglobin level, mean (SD), g/dL^b^	13.4 (2.2)	13.4 (2.1)	.95
INR, mean (SD)^c^	1.2 (0.4)	1.3 (0.7)	.048
PTT, mean (SD), s^c^	35.2 (18.5)	35.1 (21.6)	.96
Multiple trauma with ISS >15, No. (%)^d^	55 (12)	59 (12)	>.99
Maximal AIS score, mean (SD)^e^			
Head and neck^f^	2.4 (1.4)	2.3 (1.3)	.17
Face^g^	1.7 (0.8)	1.6 (0.8)	.39
Thorax^h^	2.2 (1.0)	2.4 (1.1)	.33
Abdomen^i^	2.1 (0.7)	2.4 (0.8)	.007
Extremities^j^	2.2 (0.8)	2.4 (0.9)	.049
External^k^	1.3 (0.7)	1.3 (0.7)	.80

Abbreviations: AIS, Abbreviated Injury Scale; INR, international normalized ratio; ISS, Injury Severity Score; MVC, motor vehicle crash; PTT, partial thromboplastin time; WBCT, whole-body computed tomography.

SI conversion factor: To convert hemoglobin to grams per liter, multiply by 10.0.

^a^ Based on 923 (426 and 497) patients, accounting for missing data. Calculated as heart rate in beats per minute divided by systolic blood pressure in millimeters of mercury.

^b^ Based on 968 (465 and 503) patients, accounting for missing data.

^c^ Based on 961 (459 and 502) patients, accounting for missing data.

^d^ Indicates major trauma.

^e^ Scores range from 0 to 6, with 6 indicating maximum severity.

^f^ Based on 455 (210 and 245) patients with head and neck injuries.

^g^ Based on 85 (30 and 55) patients with facial trauma.

^h^ Based on 153 (73 and 80) patients with thoracic injuries.

^i^ Based on 168 (77 and 91) patients with abdominal injuries.

^j^ Based on 228 (121 and 107) patients with fractures of the pelvis or extremities.

^k^ Based on 61 (37 and 24) patients with skin and soft tissue injuries.

The incidence of missed diagnoses fluctuated over time (eFigure 3 in the Supplement). By segmented regression analysis, the period of observation (β = −0.002; *P* = .83), implementation of the low-dose algorithm (β = 0.056; *P* = .50), and the interval after implementation (β = −0.007, *P* = .58) did not influence the risk of missed diagnoses. The Durbin-Watson statistic (*d* = 2.323) suggested no significant autocorrelation. Altogether, 109 of 468 patients in the standard-dose WBCT group (23.3%) and 107 of 503 patients in the low-dose WBCT group (21.3%) had any missed injury diagnosis at the point of care (RD, −2.0% [95% CI, −7.3% to 3.2%]; unadjusted OR, 0.89 [95% CI, 0.66-1.20]; *P* = .45). Among patients with any serious injury classified as 3 or greater on the Abbreviated Injury Scale (n = 411) (6 indicates maximum severity), 71 of 193 patients in the standard-dose WBCT group (36.8%) and 74 of 218 in the low-dose WBCT group (33.9%) had missed injury diagnoses (RD, −2.8% [95% CI, −12.1% to 6.4%]; unadjusted OR, 0.88 [95% CI, 0.59-1.32]; *P* = .55).

**Figure 2.**
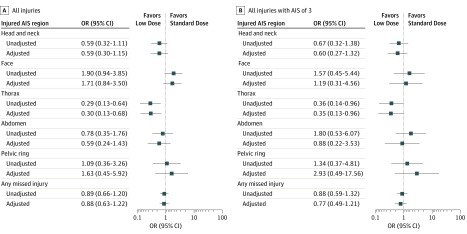
Unadjusted and Adjusted Odds Ratios (ORs) of Missed Injury Diagnoses Adjustment was made using a multivariable logistic regression model, accounting for age, sex, intubation, heart rate, systolic blood pressure, hemoglobin concentration, international normalized ratio and partial thromboplastin time on admission, a positive finding of thoracoabdominal focused ultrasonographic scan at the trauma bay, and the interval from admission to whole-body computed tomography. AIS indicates Abbreviated Injury Scale score (1 indicates minor and 6, maximum).

Multivariable logistic regression showed no differences between raw and adjusted estimates in the OR of missed injuries in different anatomical regions (Figure 2).

Ratings of subjective image quality varied across observers and region of interest (eFigure 4 in the Supplement). Both WBCT protocols showed high specificity in detecting injuries in various body areas (eTable 3 in the Supplement). Sensitivity, however, varied markedly across anatomical regions and was particularly low in case of hemothorax, hollow visceral tears, hemoperitoneum, retroperitoneal bleeding, and kidney injuries (eTable 4 in the Supplement).

**Figure 3.**
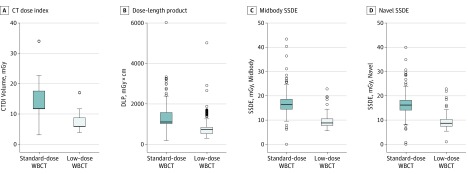
Dose Estimates of Standard-Dose and Low-Dose Whole-Body Computed Tomographic (WBCT) Scans The low-dose protocol used the iDose^4^ image processing algorithm. Data are expressed as medians and interquartile range (error bars). Circles represent outliers. For better readability, single extreme outliers are not shown for the standard-dose group (computed tomographic dose index [CTDI] volume, 1174 mGy; dose-length product [DLP] 33063.1 mGy/cm; size-specific dose estimate [SSDE] midbody, 1801.6 mGy; SSDE navel, 1823.6 mGy) or the low-dose group (CTDI volume, 585 mGy; DLP, 5021.1 mGy/cm; SSDE midbody, 891.9 mGy; SSDE navel, 866.8 mGy).

Low-dose WBCT significantly decreased exposure to radiation (Figure 3). Median volume CT dose index was reduced from 11.7 (IQR, 11.7-17.6) to 5.9 (IQR, 5.9-8.8) mGy (*P* < .001). Median DLP was reduced from 1109 (IQR, 1020-1578) to 735 (IQR, 525-847) mGy/cm (*P* < .001). Median size-specific dose estimate was reduced from 16.4 (IQR, 14.5-18.6) to 8.8 (IQR, 7.7-10.6; *P* < .001) at midbody and from 16.2 (IQR, 14.1-18.2) to 8.7 (IQR, 7.4-10.3; *P* < .001) at navel level.

The contrast-to-noise ratio consistently favored low-dose WBCT for all investigated anatomical regions (eFigure 5 in the Supplement). Median contrast-to-noise ratio was 28.9 (IQR, 16.8-43.8) vs 11.7 (IQR, 9.0-17.1) in the carotid artery (*P* < .001), 17.8 (IQR, 13.7-24.2) vs 15.1 (IQR, 10.7-19.6) in the aortic arch (*P* < .001), 2.5 (IQR, 1.4-3.5) vs 2.0 (IQR, 1.2-2.9) in the liver (*P* < .001), 9.8 (IQR, 7.2-13.2) vs 8.3 (IQR, 5.7-11.0) in the kidney (*P* < .001), 13.1 (IQR, 9.3-19.1) vs 11.6 (IQR, 7.7-16.4) in the aorta (*P* < .001), 7.4 (IQR, 5.6-9.7) vs 4.7 (IQR, 3.5-6.4) in the seventh cervical vertebral (*P* < .001), and 5.9 (IQR, 4.1-8.8) vs 4.2 (IQR, 2.8-6.5) in the first lumbar vertebral (*P* < .001). Four adverse events occurred (ie, extravasates of intravenously admitted contrast agent), with 3 (0.6%) and 1 (0.2%) incidents occurring in either group (*P* = .28). No other intervention-related events compromising patients’ safety were observed during the study.

## Discussion

In this prospective time-series cohort study, low-dose WBCT using statistical image reconstruction did not increase the risk of missed injury diagnoses at the point of care in patients with suspected blunt multiple trauma compared with standard-dose WBCT scanning. Low-dose WBCT almost halved radiation exposure and improved the contrast-to-noise ratio compared with standard-dose imaging, while maintaining diagnostic accuracy.

Conflicting evidence about the effect of primary WBCT on patient outcomes in major trauma is available. The only randomized clinical trial^12^ at present failed to show a difference in raw mortality between both diagnostic options, whereas large-scale registries suggest a significantly decreased risk-adjusted ratio of observed to expected deaths with primary WBCT.^14,37,38,39^ Excess radiation remains a major concern and obstacle to the liberal use of primary WBCT in trauma resuscitation.^14,40,41,42^

The observed rate of missed diagnoses in this study markedly exceeded that from a recent French investigation that included 2354 scans in patients with trauma obtained at 26 sites during a 5-year period (12.9%; 95% CI, 11.6%-14.3%)^43^ and other studies.^34,44^ Strict independent confirmation of positive and negative index test results may explain the rather high frequency of missed injury diagnoses compared with previous studies that calculated the risk by a secondary review of initial scans. A review of all subsequent clinical, surgical, and imaging findings was considered the most appropriate (though still imperfect) diagnostic reference test to verify initial WBCT results, because it would be unsuitable and unethical to assign patients to a second WBCT, magnetic resonance imaging, or even invasive procedures to confirm index test findings.

### Limitations

Although this study was originally planned as a noninferiority randomized clinical trial, the IRB prohibited random allocation of patients to either imaging strategy. The IRB’s ethical logic was that no previous experimental data had demonstrated that low-dose WBCT does not pose any extra risk of missed injury diagnoses to patients compared with standard-dose WBCT. The ultimate risk of a missed injury diagnosis by low-dose WBCT was considered more important than the remote lifetime attributable risk of cancer by standard-dose WBCT. The IRB approved this controlled, quasi-confirmatory before-and-after study, and several statistical efforts were made to control primary end points for time-dependent patient- and intervention-related variables. The baseline profile was well balanced among groups, and there was no marked association of injury-related or other variables with effect estimates. Conservative definitions of missed injuries were used, because blunt trauma minor injuries or their combination may be detrimental in the long term. This, however, may have led to an overestimate of the incidence of missed injuries and an underestimate of the sensitivity of WBCT with either dose protocol. Radiation exposure was determined by volume CT dose index, DLP, and size-specific dose estimate rather than effective doses. Patient- and organ-specific dose estimates depend on conversion factors specific to individual health care systems and different estimation methods (eg, Monte-Carlo simulation).^45,46^ However, volume CT dose index and DLP are valid measures to compare radiation exposure between different WBCT protocols.^31^ Although objective image quality was better with the low-dose protocol, subjective image quality varied considerably among regions of interest.

## Conclusions

The findings of this study suggest that low-dose WBCT may safely replace standard-dose WBCT in diagnostic workup of blunt multiple trauma. It provided comparable diagnostic accuracy at a much lower radiation dose and was not associated with extra harms. Because this was a quasi-experimental study, a large-scale, multicenter randomized clinical trial is warranted to confirm our findings.

## Notes

Supplement. **eTable 1.** CT Scanning Parameters **eTable 2.** Therapeutic Consequences From WBCT **eTable 3. **Diagnostic Accuracy Among Different Anatomical Regions **eTable 4. **Accuracy of WBCT in Diagnosing Individual Injuries **eFigure 1.** Meta-analysis of Studies Reporting on Missed Injuries With WBCT **eFigure 2. **Sample Size Calculation With Different Clinical Scenarios **eFigure 3.** Incidence of Missed Injury Diagnoses Over Time **eFigure 4.** Subjective Rating of Image Quality by 2 Independent Observers Using a 100-mm Visual Analog Scale (VAS) **eFigure 5.** Contrast-to-Noise Ratio (CNR) in Different Regions of Interest (ROI)
