# Supplementary material for: Association of Low-Dose Whole-Body Computed Tomography With Missed Injury Diagnoses and Radiation Exposure in Patients With Blunt Multiple Trauma
Source: JAMA Surg. 2020 Jan 15;155(3):224–32. doi: 10.1001/jamasurg.2019.5468 (PMC6990738; doi:10.1001/jamasurg.2019.5468)
Supplement: Supplement. — eTable 1. CT Scanning Parameters eTable 2. Therapeutic Consequences From WBCT eTable 3. Diagnostic Accuracy Among Different Anatomical Regions eTable 4. Accuracy of WBCT in Diagnosing Individual Injuries eFigure 1. Meta-analysis of Studies Reporting on Missed Injuries With WBCT eFigure 2. Sample Size Calculation With Different Clinical Scenarios eFigure 3. Incidence of Missed Injury Diagnoses Over Time eFigure 4. Subjective Rating of Image Quality by 2 Independent Observers Using a 100-mm Visual Analog Scale (VAS) eFigure 5. Contrast-to-Noise Ratio (CNR) in Different Regions of Interest (ROI) [file jamasurg-155-224-s001.pdf]

Supplementary Online Content

Stengel D, Mutze S, Güthoff C, et al. Association of low-dose whole-body computed tomography with missed injury diagnoses and radiation exposure in patients with blunt multiple trauma. *JAMA Surg.* Published online January 15, 2020. doi:10.1001/jamasurg.2019.5468

eTable 1. CT Scanning Parameters

eTable 2. Therapeutic Consequences From WBCT

eTable 3. Diagnostic Accuracy Among Different Anatomical Regions

eTable 4. Accuracy of WBCT in Diagnosing Individual Injuries

eFigure 1. Meta-analysis of Studies Reporting on Missed Injuries With WBCT

eFigure 2. Sample Size Calculation With Different Clinical Scenarios

eFigure 3. Incidence of Missed Injury Diagnoses Over Time

eFigure 4. Subjective Rating of Image Quality by 2 Independent Observers Using a 100-mm Visual Analog Scale (VAS)

eFigure 5. Contrast-to-Noise Ratio (CNR) in Different Regions of Interest (ROI)

This supplementary material has been provided by the authors to give readers additional information about their work.

© 2020 Stengel D et al. *JAMA Surg.*

eTable 1. CT Scanning Parameters

Parameter	Standard-Dose WBCT	Low-Dose WBCT
Slice thickness, mm		
Axial	5.0 (0.20) + 0.6 (0.02)	5.0 (0.20) + 0.6 (0.02)
Coronal and sagittal reconstruction	3.0 (0.12)	3.0 (0.12)
BMI <35		
Tube voltage, kVp	120	120
Effective tube current rotation time normalized to helical pitch, mAs	180	90
Pitch factor	0.9	0.9
Reconstructed slice thickness, mm	128 x 0.625	128 x 0.625
Reconstruction kernel	C	D
BMI ≥35		
Tube voltage, kVp	120	120
Effective tube current rotation time normalized to helical pitch, mAs	270	135
Pitch factor	0.9	0.9
Reconstructed slice thickness, mm	128 x 0.625	128 x 0.625
Reconstruction kernel	C	C

Data are mean (SD). Tube settings include peak kilovoltage (kVP) and milliampere-seconds (mAs). BMI denotes Body Mass Index.

© 2020 Stengel D et al. *JAMA Surg.*

eTable 2. Therapeutic Consequences From WBCT

	No. (% [95% CI]) of Patients									
	Standard-dose WBCT (n = 468)					Low-dose WBCT (n = 503)				
Consequence	AIS Region					AIS Region				
	Head and Neck	Face	Thorax	Abdomen	Pelvic Ring	Head and Neck	Face	Thorax	Abdomen	Pelvic Ring
	111	124	116	90	44	114	133	134	97	40
Immediate surgery	44 (40 [30 to 49])	39 (31 [23 to 40])	43 (37 [28 to 47])	28 (31 [22 to 42])	16 (36 [22 to 52])	38 (33 [25 to 43])	32 (24 [17 to 32])	43 (32 [24 to 41])	25 (26 [17 to 36])	10 (25 [13 to 41])
Admission to ICU	45 (41 [31 to 50])	51 (41 32 to 50])	39 (34 [25 to 43])	38 (42 [32 to 53])	22 (50 [35 to 65])	52 (46 [36 to 55])	58 (44 [35 to 52])	55 (41 [33 to 50])	38 (39 [29 to 50])	24 (60 [43 to 75])
Admission to ward	22 (20 [13 to 28])	30 (24 17 to 33])	33 (28 [20 to 38]))	24 (27 [18 to 37])	6 (14 [5 to 27])	24 (21 [14 to 30])	41 (31 [23 to 39])	32 (24 [17 to 32])	33 (34 [25 to 44])	6 (15 [6 to 30])
Discharge	0 (0 [0 to 0])	4 (3 [1 to 8])	1 (1) [0 to 1])	0 (0 [0 to 0])	0 (0 [0 to 1])	0 (0 [0 to 0])	2 (2 [0 to 5])	4 (3 [1 to 7])	1 (1 [0 to 6])	0 (0 [0 to 9])

© 2020 Stengel D et al. *JAMA Surg.*

eTable 3. Diagnostic Accuracy Among Different Anatomical Regions

	Standard-Dose WBCT			Low-Dose WBCT		
**AIS region**	**Prevalence, %**	**Sensitivity, % (95% CI)**	**Specificity, % (95% CI)**	**Prevalence, %**	**Sensitivity, % (95% CI)**	**Specificity, % (95% CI)**
Head and Neck	26.7	79.2 (71.0 to 85.9)	96.5 (94.0 to 98.2)	22.9	85.2 (77.4 to 91.1)	95.9 (93.4 to 97.6)
Face	13.7	81.3 (69.5 to 89.9)	82.2 (78.1 to 85.8)	17.5	72.7 (62.2 to 81.7)	83.4 (79.4 to 86.8)
Thorax	26.3	79.7 (71.5 to 86.4)	94.8 (91.9 to 96.9)	25.5	93.8 (88.1 to 97.3)	96.3 (93.8 to 97.9)
Abdomen	17.3	84.0 (74.1 to 91.2)	94.1 (91.2 to 96.2)	17.9	87.8 (79.2 to 93.7)	95.6 (93.2 to 97.4)
Pelvic Ring (Extremities)	9.0	85.7 (71.5 to 94.6)	98.1 (96.3 to 99.2)	9.1	84.8 (71.1 to 93.7)	99.8 (98.8 to 100)

© 2020 Stengel D et al. *JAMA Surg.*

eTable 4. Accuracy of WBCT in Diagnosing Individual Injuries

	Standard-dose WBCT			Low-dose WBCT		
**Injury**	**Prevalence, %**	**Sensitivity, % (95% CI)**	**Specificity, % (95% CI)**	**Prevalence, %**	**Sensitivity, % (95% CI)**	**Specificity, % (95% CI)**
Skull fractures	8.5	80.0 (64.4 to 90.9)	96.7 (94.6 to 98.2)	7.6	86.8 (71.9 to 95.6)	99.4 (98.1 to 99.9)
Brain injury	16.7	82.1 (71.7 to 89.8)	97.4 (95.3 to 98.8)	15.7	86.1 (76.5 to 92.8)	98.3 (96.6 to 99.3)
BCVI	2.4	72.7 (39.0 to 94.0)	98.5 (96.9 to 99.4)	1.2	100.0 (54.1 to 100.0)	98.8 (97.4 to 99.6)
Cervical spine injury	10.7	88.0 (75.7 to 95.5)	98.8 (97.2 to 99.6)	7.2	80.6 (64.0 to 91.8)	98.3 (96.7 to 99.3)
Thoracic spine injury	12.6	78.0 (65.3 to 87.7)	97.8 (95.9 to 99.0)	9.3	93.6 (82.5 to 98.7)	98.7 (97.2 to 99.5)
Serial rib fractures	12.0	75.0 (61.6 to 85.6)	99.0 (97.5 to 99.7)	13.1	83.3 (72.1 to 91.4)	98.6 (97.0 to 99.5)
Lung contusion	4.9	69.6 (47.1 to 86.8)	96.4 (94.2 to 97.9)	9.7	63.3 (48.3 to 76.6)	97.4 (95.4 to 98.6)
Pneumothorax	9.6	84.4 (70.5 to 93.5)	97.9 (96.0 to 99.0)	9.5	83.3 (69.8 to 92.5)	98.7 (97.2 to 99.5)
Hemothorax	3.4	31.3 (11.0 to 58.7)	96.2 (94.0 to 97.8)	4.0	40.0 (19.1 to 63.9)	99.2 (97.9 to 99.8)
Aortic or cardiac tear	1.5	42.9 (9.9 to 81.6)	100.0 (99.2 to 100.0)	0.6	66.7 (9.4 to 99.2)	100.0 (99.3 to 100.0)
Lumbar spine injury	14.3	86.6 (76.0 to 93.7)	96.5 (94.2 to 98.1)	13.1	93.9 (85.2 to 98.3)	96.1 (93.8 to 97.7)
Hepatic rupture	0.6	0.0 (0.0 to 70.8)	99.6 (98.5 to 99.9)	2.6	69.2 (38.6 to 90.9)	99.8 (98.9 to 100.0)
Splenic rupture	2.6	85.7 (57.2 to 98.2)	98.7 (97.1 to 99.5)	3.4	64.7 (38.3 to 85.8)	99.8 (98.9 to 100.0)
Hollow visceral tear	1.1	80.0 (28.4 to 99.5)	98.7 (97.2 to 99.5)	1.2	0.0 (0.0 to 45.9)	99.2 (98.0 to 99.8)
Hemoperitoneum	2.6	50.0 (21.1 to 78.9)	99.1 (97.8 to 99.8)	4.2	28.6 (11.3 to 52.2)	99.4 (98.2 to 99.9)
Retroperitoneum	1.7	50.0 (15.7 to 84.3)	95.9 (93.6 to 97.5)	3.0	33.3 (11.8 to 61.6)	98.0 (96.3 to 99.0)
Kidney laceration	0.6	33.3 (0.8 to 90.6)	99.4 (98.1 to 99.9)	1.8	44.4 (13.7 to 78.8)	100.0 (99.3 to 100.0)
Pelvic fracture	8.3	89.7 (75.8 to 97.1)	98.6 (97.0 to 99.5)	8.2	80.5 (65.1 to 91.2)	99.4 (98.1 to 99.9)
Acetabular fracture	3.6	82.4 (56.6 to 96.2)	98.9 (97.4 to 99.6)	3.6	83.3 (58.6 to 96.4)	99.8 (98.9 to 100.0)

BCVI denotes blunt carotid and vertebral vessel injury

© 2020 Stengel D et al. *JAMA Surg.*

eFigure 1. Meta-analysis of Studies Reporting on Missed Injuries With WBCT

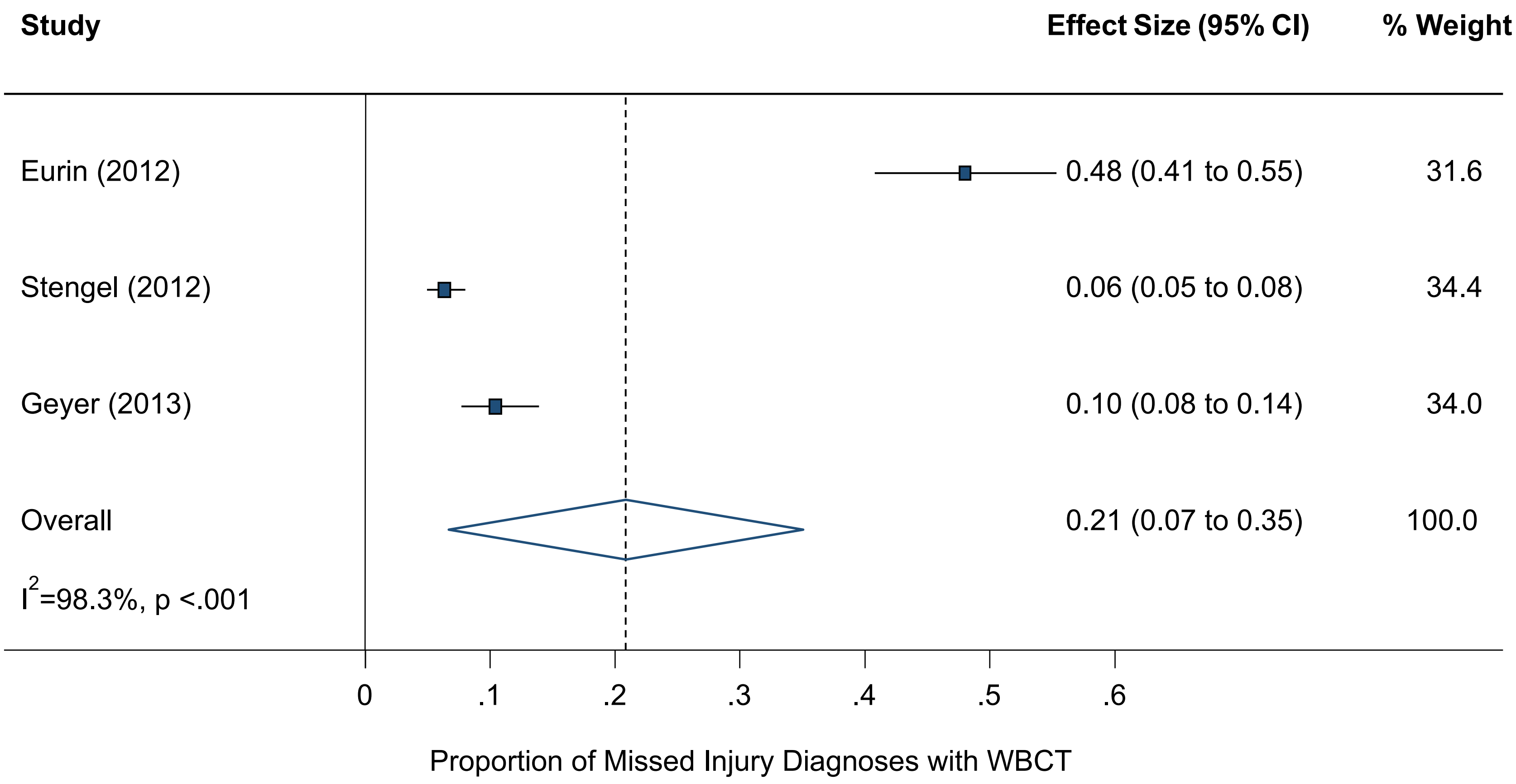

© 2020 Stengel D et al. *JAMA Surg.*

eFigure 2. Sample Size Calculation With Different Clinical Scenarios

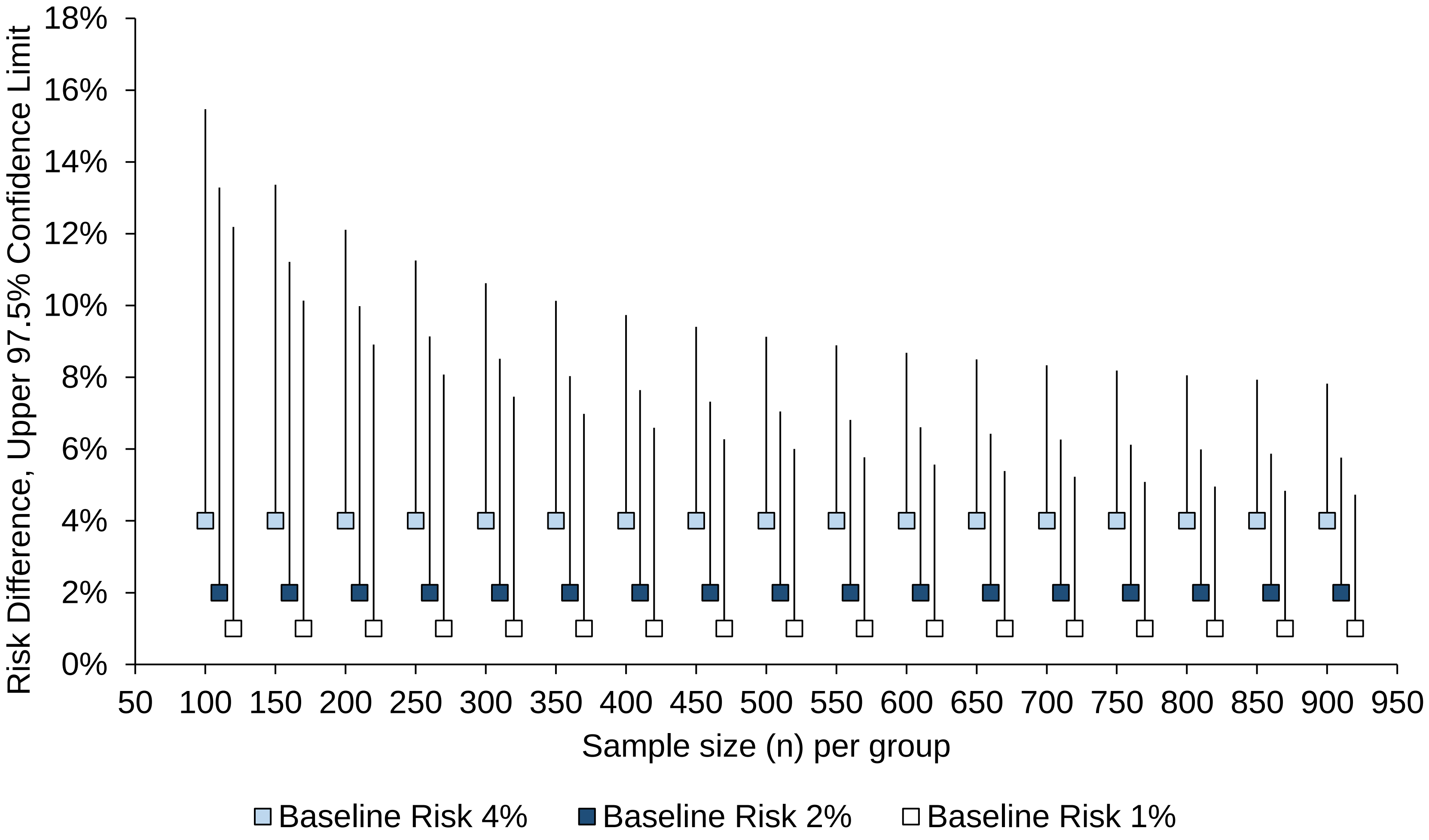

© 2020 Stengel D et al. *JAMA Surg.*

eFigure 3. Incidence of Missed Injury Diagnoses Over Time

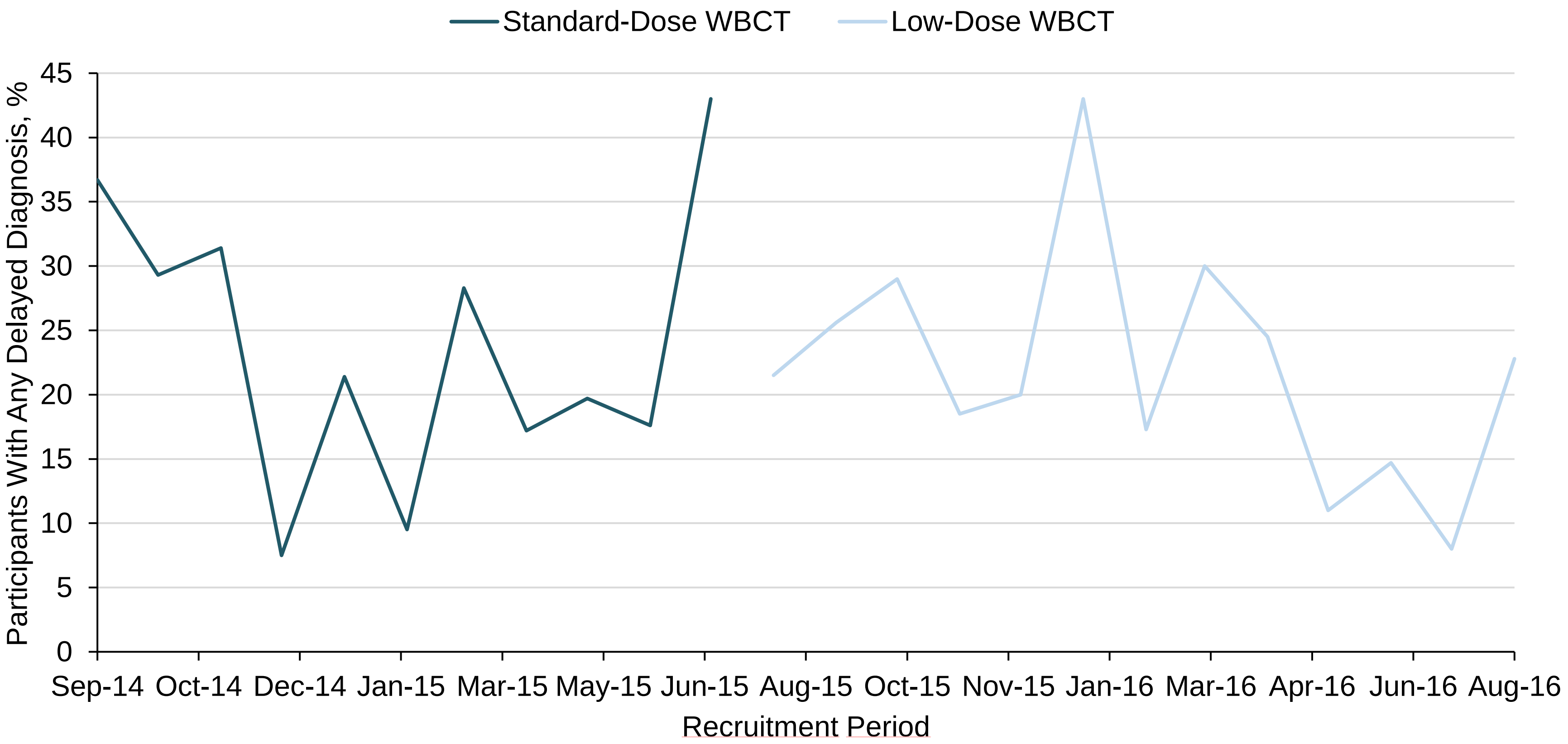

© 2020 Stengel D et al. *JAMA Surg.*

eFigure 4. Subjective Rating of Image Quality by 2 Independent Observers Using a 100-mm Visual Analog Scale (VAS)

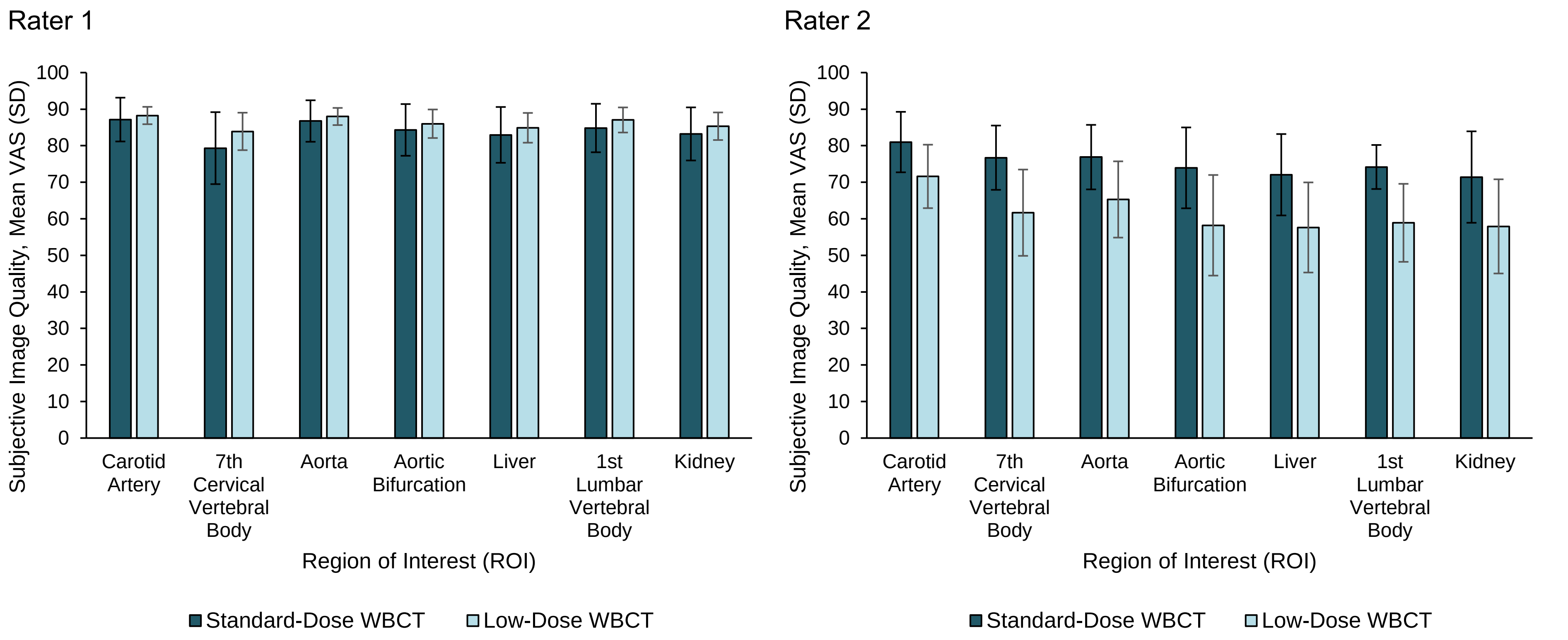

© 2020 Stengel D et al. *JAMA Surg.*

eFigure 5. Contrast-to-Noise Ratio (CNR) in Different Regions of Interest (ROI)

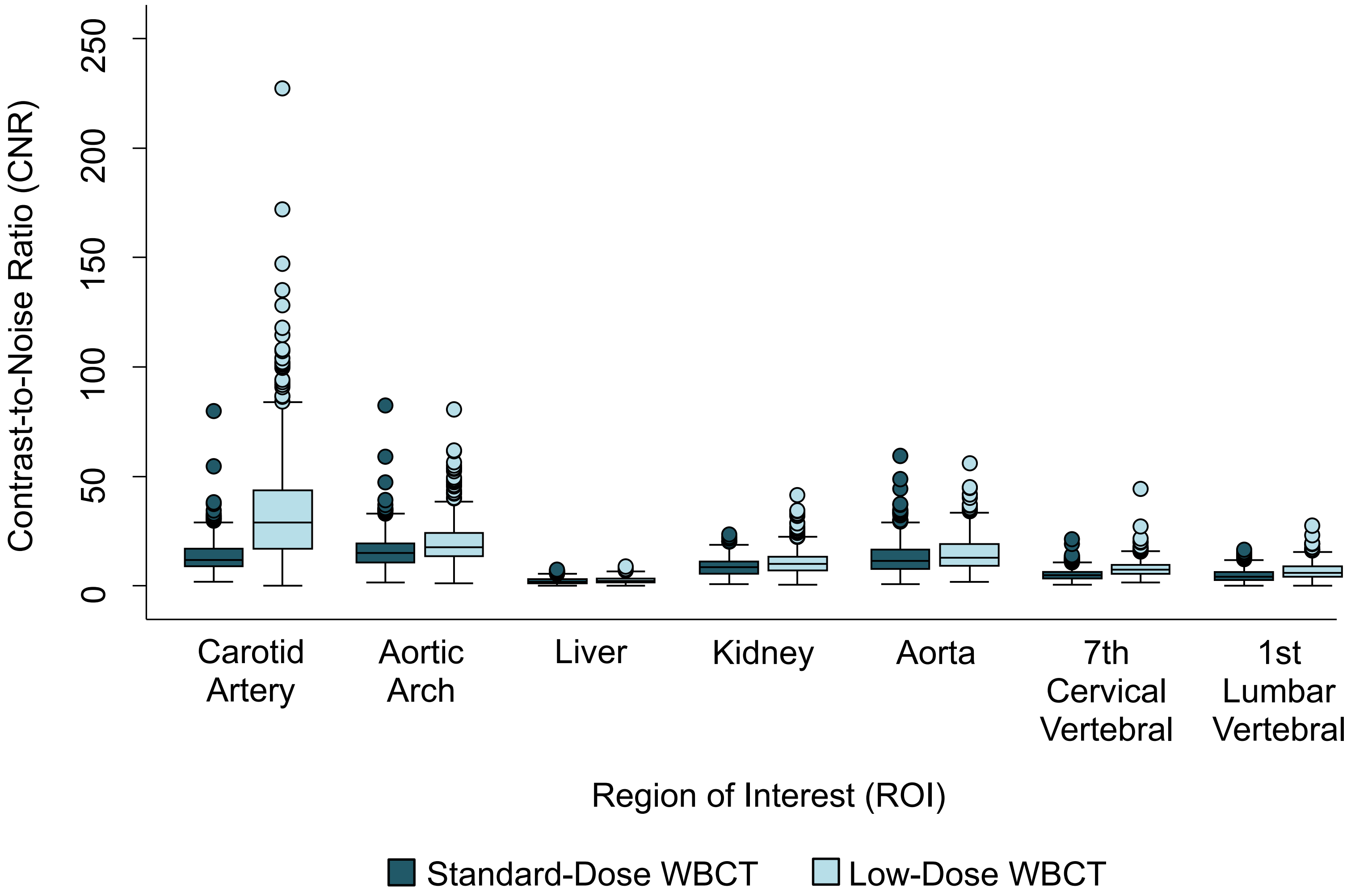

© 2020 Stengel D et al. *JAMA Surg.*
